# Splenectomy Correlates With Increased Risk of Acute Pancreatitis: A Case-Control Study in Taiwan

**DOI:** 10.2188/jea.JE20150214

**Published:** 2016-09-05

**Authors:** Shih-Wei Lai, Cheng-Li Lin, Kuan-Fu Liao

**Affiliations:** 1 College of Medicine, China Medical University, Taichung, Taiwan; 2 Department of Family Medicine, China Medical University Hospital, Taichung, Taiwan; 3 Management Office for Health Data, China Medical University Hospital, Taichung, Taiwan; 4 College of Medicine, Tzu Chi University, Hualien, Taiwan; 5 Department of Internal Medicine, Taichung Tzu Chi General Hospital, Taichung, Taiwan; 6 Graduate Institute of Integrated Medicine, China Medical University, Taichung, Taiwan

**Keywords:** acute pancreatitis, alcohol-related disease, biliary stone, diabetes mellitus, splenectomy

## Abstract

**Objective:**

The objective of the study was to investigate the association between splenectomy and acute pancreatitis.

**Methods:**

We conducted a case-control study using the database of the Taiwan National Health Insurance Program. We included 7666 subjects aged 20–84 years with first-time acute pancreatitis during the period of 1998–2011 as cases and 30 664 randomly selected subjects without acute pancreatitis as controls. Both cases and controls were matched for sex, age, and index year of acute pancreatitis diagnosis. The association of acute pancreatitis with splenectomy was examined using a multivariable unconditional logistic regression model and reported as an odds ratio and its 95% confidence interval (CI).

**Results:**

After adjustment for covariables, the adjusted odds ratio of acute pancreatitis was 2.90 for subjects with splenectomy (95% CI, 1.39–6.05) compared with subjects without splenectomy.

**Conclusions:**

Splenectomy is associated with acute pancreatitis. Further studies are necessary to clarify the underlying mechanism.

## INTRODUCTION

The immunologic and hematologic functions of the spleen in humans are well known. Specifically, the spleen protects against infections mediated by innate and adaptive immunity.^1^^,^^2^ Further, in persons with splenectomy, there is growing evidence of serious post-splenectomy complications, including atelectasis, pulmonary embolism and bleeding in the early period, and pulmonary tuberculosis and overwhelming postsplenectomy infections in the late period^1^^–^^6^; however, acute pancreatitis has not been well studied.

Acute pancreatitis is a serious disease due to its high morbidity and mortality worldwide. To date, many risk factors associated with acute pancreatitis have been well established, including alcoholism, biliary stones, cardiovascular disease, diabetes mellitus, hepatitis B infection, hepatitis C infection, and hypertriglyceridemia^7^^–^^10^; however, 9% to 36.6% of acute pancreatitis patients remained idiopathic in different studies.^8^^,^^11^

To date, no systematic study has investigated the association between splenectomy and acute pancreatitis. Whether splenectomy has a positive or negative effect on risk of acute pancreatitis is unknown. As mentioned above, we rationally hypothesize that the pancreas might be easily infected by offending microorganisms involved in overwhelming post-splenectomy infections, which might subsequently lead to pancreatic inflammation. A better understanding of the relationship between splenectomy and acute pancreatitis may support interventions such as pneumococcal vaccination in persons with splenectomy. Therefore, we conducted a case-control study using the nationwide database of the Taiwan National Health Insurance Program to investigate this issue.

## METHODS

### Data source

This was a nationwide case-control study using the database of the Taiwan National Health Insurance Program. Briefly, this insurance program began in March 1, 1995, and covers about 99% of the 23 million persons living in Taiwan.^12^ The details of the program have been thoroughly addressed in previous studies.^13^^–^^15^ This study was approved by the Ethics Review Board of China Medical University and Hospital in Taiwan (CMUH-104-REC2-115).

### Identification of cases and controls

We selected subjects aged 20–84 years with first-time acute pancreatitis during the period of 1998–2011 as the case group according to the International Classification of Diseases 9th Revision (ICD-9 code 577.0). To increase statistical power, for each case of acute pancreatitis, four subjects without acute pancreatitis were randomly selected from the same database as the control group. Both groups were matched for sex, age (within 5 years), and index year of acute pancreatitis diagnosis. The index date for each case was defined as the date of acute pancreatitis diagnosis. The index date for control subjects was a randomly assigned day and month with the same index year of the corresponding case. To diminish biased results, subjects undergoing splenectomy within 1 month of acute pancreatitis diagnosis were excluded from the study. Subjects with prior diagnosis of chronic pancreatitis (ICD-9 code 577.1) or pancreatic cancer (ICD-9 code 157) before the date of acute pancreatitis diagnosis were also excluded from the study.

### Comorbidities potentially associated with acute pancreatitis

Comorbidities diagnosed before the date of acute pancreatitis diagnosis that were potentially associated with acute pancreatitis were included in the study as follows: splenectomy (ICD-9 procedure code 41.5); alcohol-related disease (ICD-9 codes 291, 303, 305.00, 305.01, 305.02, 305.03, 790.3, and V11.3); biliary stone (ICD-9 code 574); cardiovascular disease, including coronary artery disease, heart failure, cerebrovascular disease, and peripheral atherosclerosis (ICD-9 codes 410–414, 428, 430–438, and 440–448); chronic kidney disease (ICD-9 codes 585–586 and 588.8–588.9); chronic obstructive pulmonary disease (ICD-9 codes 491, 492, 493, and 496); diabetes mellitus (ICD-9 code 250); hepatitis B (ICD-9 codes V02.61, 070.20, 070.22, 070.30, and 070.32); hepatitis C (ICD-9 codes V02.62, 070.41, 070.44, 070.51, and 070.54); hypercalcemia (ICD-9 code 275.42); hyperparathyroidism (ICD-9 code 252.0); and hypertriglyceridemia (ICD-9 code 272.1). Smoking, which has been found to be associated with acute pancreatitis,^16^ was not recorded in this database. Therefore, chronic obstructive pulmonary disease was used as an alternative variable. Measures related to socioeconomic status, such as income, education, and occupation, were not recorded in this database and could not be included in the study. On the basis of ICD-9 codes, the diagnosis accuracy of included comorbidities has been reviewed in previous high-quality studies.^17^^–^^20^ Few patients having acute pancreatitis and/or the comorbidities studied would be expected to never go to the hospital for treatment. In order to avoid subjects who were coded by mistake or diagnosed inaccurately and to enhance the diagnosis validity, acute pancreatitis and comorbidities were identified with at least two consensus diagnoses in ambulatory care and/or during hospitalization.

### Statistical analysis

The Chi-square test and fisher-exact test for categorical variables and *t*-test for continuous variables were used to compare the differences between the case group and the control group for distributions of demographic factors and comorbidities. All variables were first examined in a univariable unconditional logistic regression model. Those found to be significant in the univariable unconditional logistic regression model were then included in the multivariable unconditional logistic regression model, and odds ratios (ORs) and 95% confidence intervals (CIs) for the association of acute pancreatitis with splenectomy and other comorbidities were calculated. The probability value <0.05 was considered statistically significant (SAS software version 9.2, SAS Institute Inc., Cary, NC, USA).

## RESULTS

### Characteristics of the study population

Table 1 presents the characteristics of the study population. There were 7666 subjects in the case group and 30 664 subjects in the control group, with similar distributions of sex and age. There were 3258 subjects (42.5%) without comorbidities in the case group and 21 429 subjects (69.9%) without comorbidities in the control group. Splenectomy, alcohol-related disease, biliary stone, cardiovascular disease, chronic kidney disease, chronic obstructive pulmonary disease, diabetes mellitus, hepatitis B, hepatitis C, hyperparathyroidism, and hypertriglyceridemia were more prevalent in the case group than the control group (Chi-square test, *P* < 0.001 for all). The mean (standard deviation) ages were 50.4 (15.8) years in the case group and 50.0 (16.0) years in the control group (*t*-test, *P* = 0.05).

**Table 1.** Descriptive characteristics of cases with acute pancreatitis and controls

Characteristic	Controls *n* = 30 664		Cases *n* = 7666		*P* value^a^
	
	*n*	(%)	*n*	(%)	
Sex					0.99
Female	10 216	33.3	2554	33.3	
Male	20 448	66.7	5112	66.7	
Age group, years					0.99
20–39	9404	30.7	2351	30.7	
40–64	14 560	47.5	3640	47.5	
65–84	6700	21.9	1675	21.9	
Mean (SD) age, years^b^	50.0	16.0	50.4	15.8	0.05
Comorbidities before index date					
Splenectomy	19	0.06	15	0.20	<0.001
Alcohol-related disease	112	0.37	339	4.42	<0.001
Biliary stone	602	1.96	1585	20.7	<0.001
Cardiovascular disease	5143	16.8	1946	25.4	<0.001
Chronic kidney disease	489	1.59	338	4.41	<0.001
Chronic obstructive pulmonary disease	3622	11.8	1217	15.9	<0.001
Diabetes mellitus	3074	10.0	1539	20.1	<0.001
Hepatitis B	546	1.78	318	4.15	<0.001
Hepatitis C	293	0.96	191	2.49	<0.001
Hypercalcemia^c^	6	0.02	3	0.04	0.18
Hyperparathyroidism	22	0.07	19	0.25	<0.001
Hypertriglyceridemia	168	0.55	111	1.45	<0.001

SD, standard deviation.

Data are presented as the number of subjects in each group with percentages given in parentheses, or mean with standard deviation given in parentheses.

^a^Chi-square test comparing subjects with and without acute pancreatitis.

^b^*t*-test comparing subjects with and without acute pancreatitis.

^c^Fisher’s exact test comparing subjects with and without acute pancreatitis.

### Odds ratio of acute pancreatitis associated with splenectomy and other comorbidities

Table 2 presents the ORs of acute pancreatitis associated with splenectomy and other comorbidities. After controlling for covariables, the adjusted OR of acute pancreatitis was 2.90 (95% CI, 1.39–6.05) for subjects with splenectomy compared with subjects without splenectomy. Alcohol-related disease, biliary stone, cardiovascular disease, chronic kidney disease, diabetes mellitus, hepatitis B, hepatitis C, hyperparathyroidism, and hypertriglyceridemia were also significantly related to acute pancreatitis.

**Table 2.** Crude and adjusted odds ratios and 95% confidence intervals of acute pancreatitis associated with splenectomy and other comorbidities

Variable	Crude		Adjusted^a^	
	
	OR	(95% CI)	OR	(95% CI)
Sex, male vs female	1.00	(0.95, 1.06)	—	—
Age, per one year	1.00	(0.99, 1.00)	—	—
Comorbidities before index date, yes vs no				
Splenectomy	3.16	(1.61, 6.23)	2.90	(1.39, 6.05)
Alcohol-related disease	12.6	(10.2, 15.6)	14.8	(11.9, 18.5)
Biliary stone	13.0	(11.8, 14.4)	12.5	(11.3, 13.8)
Cardiovascular disease	1.69	(1.59, 1.79)	1.10	(1.02, 1.18)
Chronic kidney disease	2.85	(2.47, 3.28)	2.04	(1.74, 2.40)
Chronic obstructive pulmonary disease	1.41	(1.31, 1.51)	1.02	(0.94, 1.11)
Diabetes mellitus	2.25	(2.11, 2.41)	1.89	(1.75, 2.05)
Hepatitis B	2.39	(2.07, 2.75)	2.00	(1.71, 2.34)
Hepatitis C	2.65	(2.20, 3.18)	1.63	(1.32, 2.01)
Hypercalcemia	2.01	(0.50, 8.02)	—	—
Hyperparathyroidism	3.46	(1.87, 6.40)	2.21	(1.13, 4.30)
Hypertriglyceridemia	2.67	(2.10, 3.39)	2.08	(1.60, 2.70)

CI, confidence interval; OR, odds ratio.

^a^Variables found to be significant in the univariable unconditional logistic regression model were further included in the multivariable unconditional logistic regression model. ORs were additionally adjusted for alcohol-related disease, biliary stone, cardiovascular disease, chronic kidney disease, chronic obstructive pulmonary disease, diabetes mellitus, hepatitis B, hepatitis C, hyperparathyroidism, and hypertriglyceridemia.

### Risk of acute pancreatitis stratified by splenectomy and alcohol-related disease or biliary stone

Table 3 presents the risk of acute pancreatitis stratified by splenectomy and alcohol-related disease or biliary stone. Compared to subjects without splenectomy and without alcohol-related disease or biliary stone, the adjusted OR of acute pancreatitis was 3.57 (95% CI, 1.69–7.56) in those with splenectomy and without alcohol-related disease or biliary stone. The adjusted OR of acute pancreatitis was 7.93 (95% CI, 1.31–48.1) in those with splenectomy and with alcohol-related disease or biliary stone.

**Table 3.** Risk of acute pancreatitis stratified by splenectomy and alcohol-related disease and biliary stone

Variable			Adjusted OR^a^	(95% CI)

Splenectomy	Presence of alcohol-related disease or biliary stone	Case number/ control number		
No	No	5750/29 936	1.00	(Reference)
Yes	No	12/17	3.57	(1.69, 7.56)
Yes	Yes	3/2	7.93	(1.31, 48.1)

CI, confidence interval; OR, odds ratio.

^a^Adjusted for cardiovascular disease, chronic kidney disease, chronic obstructive pulmonary disease, diabetes mellitus, hepatitis B, hepatitis C, hyperparathyroidism, and hypertriglyceridemia.

## DISCUSSION

In this case-control study, we noticed that splenectomy was associated with increased odds of acute pancreatitis (adjusted OR 2.9). A review by Sinwar found that the duration between splenectomy and onset of overwhelming post-splenectomy infection could range from less than 1 week to more than 20 years.^21^ To diminish biased results, we excluded patients who underwent splenectomy within 1 month of acute pancreatitis diagnosis to ensure that splenectomy truly preceded the onset of acute pancreatitis.

Biliary stone and alcoholism are the two most common causes of acute pancreatitis. We found that alcohol-related disease and biliary stone are strongly related to acute pancreatitis (adjusted OR 14.8 for alcohol-related disease and OR 12.5 for biliary stone; Table 2). Even after adjustment for other comorbidities, the effect of alcohol-related disease and biliary stone on the OR associated with splenectomy could remain. Therefore, we conducted an additional analysis to estimate the risk of acute pancreatitis stratified by splenectomy and the presence of alcohol-related disease or biliary stone. The additional analysis revealed that, even among patients without alcohol-related disease or biliary stone, splenectomy alone was still associated with increased odds of acute pancreatitis (adjusted OR 3.57; Table 3). While the overall sample was large, the number of the subjects who underwent splenectomy was rather small (19 controls and 15 cases), which may limit the reliability of the present study results.

The pathogenetic mechanism linking splenectomy and acute pancreatitis cannot be determined in our observational study, and we did not identify other studies that can be compared with ours. To date, there is little evidence to support the association of splenectomy and acute pancreatitis. We reviewed the relevant literature to explain the biological plausibility of our findings. As we know, the spleen protects humans against infections mediated by innate and adaptive immunity.^1^^,^^2^ After splenectomy, the impaired immune functions place subjects at high risk for overwhelming postsplenectomy infections.^1^^–^^6^ Therefore, the pancreas might be easily infected by the offending microorganisms, subsequently leading to pancreatic inflammation. Splenectomy has also been found to be associated with an increased risk of type 2 diabetes mellitus,^22^ and patients with type 2 diabetes mellitus are at an elevated risk of acute pancreatitis.^10^ Whether splenectomy causes acute pancreatitis cannot be determined in an observational study. However, we think that there could be a link between splenectomy, type 2 diabetes mellitus, and acute pancreatitis.

There are some limitations inherent to this database. First, results of analysis of the database should be interpreted cautiously because the database did not have enough information on the etiologies of acute pancreatitis and splenectomy. In this study, only about 25% of acute pancreatitis patients had either alcohol-related disease or biliary stone (Table 1), so the exact etiologies of acute pancreatitis were not known in 75% of the cases, although association with some diseases was noted. Missing information about the etiologies of acute pancreatitis in the database might have introduced bias in the results. Whether acute pancreatitis is associated with overwhelming postsplenectomy infections or another unknown etiology remains inconclusive. Similarly, this database did not have enough information on the grades of acute pancreatitis, so we were unable to investigate the association between splenectomy and the grades of acute pancreatitis. Second, the indications for splenectomy were not recorded in this database. Splenectomy is commonly performed in certain disorders and diseases, such as hematological disorders, gastric cancer, or trauma, so these background conditions might confound the results. Table 1 presents 15 patients having splenectomy and acute pancreatitis. No hematological malignancies were found among these patients. Similarly, we do not know the reasons for splenectomy in the controls. Whether the reasons for splenectomy are associated with acute pancreatitis cannot be clarified in this study. Third, splenectomy predisposes patients to bacterial infection, particularly pneumococcal episodes. Whether pneumococcal vaccination could decrease the risk of acute pancreatitis among patients with splenectomy remains unclear. Fourth, the small number of cases included limits interpretability of the present results. Prospective studies with large numbers of splenectomy cases are required to confirm our findings.

The main strength of our study is that this is the first original holistic study on the association between splenectomy and acute pancreatitis. Although the underlying mechanism linking splenectomy and acute pancreatitis cannot be completely determined, our findings are clinically important.

We conclude that splenectomy is associated with acute pancreatitis. Further studies to investigate the underlying mechanism are needed.
